# Impact of Mechanical Processes as a Pre-Sulphonitriding Treatment on Tribology Properties of Selected P/M Tool Steels

**DOI:** 10.3390/ma12203431

**Published:** 2019-10-20

**Authors:** Daniel Toboła

**Affiliations:** Łukasiewicz Research Network—Institute of Advanced Manufacturing Technology, Wrocławska 37A Str., 30-011 Krakow, Poland; daniel.tobola@ios.krakow.pl; Tel.: +48-126-317-251

**Keywords:** sulfonitriding, tool steel, wear resistance, slide diamond burnishing, grinding

## Abstract

We have evaluated phase composition changes in the surface layer (SL) and wear resistance of steels investigated after various mechanical processes such as a pre-sulphonitriding treatments. Two various paths of surface modification were employed: Grinding–sulphonitriding (G-SN) and hard turning–slide burnishing–sulphonitriding (T-B-SN). Studies were carried out on Vanadis 8 and Vancron 40 tool steels, which are classified as advanced powder metallurgy (P/M) high-alloyed steels with different types and amounts of carbides. Heat treatment to the final hardness of 64 ± 1 HRC (Vanadis 8) and 62 ± 1 HRC (Vancron 40) was performed in vacuum furnaces with gas quenching. Precipitation of different types such as sulfides, nitrides, and carbides was observed using X-ray diffraction analysis. Tribological properties of SL were evaluated by pin-on-disc experiments. Pins of Al_2_O_3_ and 19MnB4 steel were used as counterbodies materials. 3D surface geometrical structure measurements were also performed. Wear tracks and cross-sections of SL were observed using optical and scanning electron microscopy. The three-stage process increases the wear resistance about 37% and 30%, respectively for Vanadis 8 and Vancron 40 (in case of alumina pins), whereas values of wear rates after tests performed against steel pins were very similar for two compared processes for both steels.

## 1. Introduction

Mechanical processes including grinding, hard turning, and burnishing belong to finishing operations, which are mainly intended to form surface layer (SL) properties e.g., surface roughness, residual stresses [1,2,3,4,5,6,7]. In traditional manufacturing processes, the finishing operation of tools and machine parts, of which hardness after heat treatment is higher than 45 HRC and even 60 HRC, is often grinding. For several decades in many industries, e.g., automotive, bearing, molds and dyes manufacturing, processes of grinding are gradually replaced by so-called “hard machining” (HM). Not always, however, does HM meet the requirements for the expected surface quality. The method to overcome these technological barriers is burnishing, which is applied both to the turned and milled surfaces [8].

Prevey [9] reports that static burnishing (e.g., rolling and sliding burnishing) provides significantly improved properties of SL compared to dynamic burnishing (shot peening). Static burnishing enables control of the depth of plastic deformation, while shot peening generates a large dislocation density in the SL due to the high degree of work-hardening, which results in unstable residual stress state. Sliding burnishing, whose specificity is the use of tools made of diamond or of similar superhard materials with a very low sliding friction on metals, may be used for treatment of the hardest steels and alloys [10].

Thermo-chemical treatment, such as nitriding or sulphonitriding, is another type of operation widely used in industrial conditions based on the diffusion change of the chemical composition of SL in order to obtain the required functional properties of a workpiece [11,12,13,14,15]. In this process, a nitrided layer is achieved, as in the case of the classical nitriding method. However, it should be noted that in the ε zone, iron sulphides are produced, which are decisive at the exploitation stage since they separate the friction surfaces and reduce their adhesion. This allows one to obtain low friction as well as high wear and abrasion resistance. This type of chemical heat treatment gives the opportunity to modify selected properties of SL in a wide range by controlling the process parameters. Production of the right amount of iron sulphide in the ε phase is extremely important in this case. Gas sulfonitriding is the final process, and therefore parts are not subjected to any additional mechanical treatment. The thickness of such layers is usually from 0.1 mm to 0.4 mm, depending on the type of material, process parameters, and application of these elements. The method of gas sulfonitriding can be used to treated elements made of low-, medium-, and high-alloy steels as well as tool steels.

**Figure 1:**
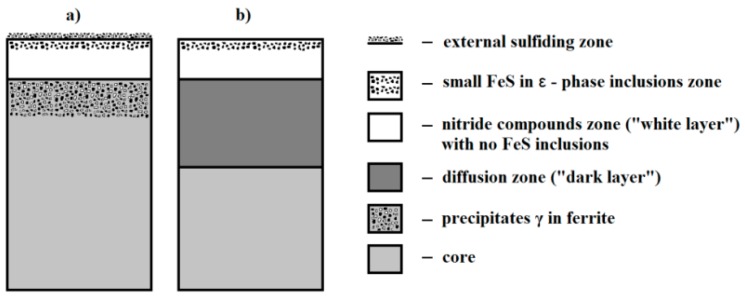
Scheme of surface layer (SL) structure on the parts after gas sulfonitriding for: (**a**) Carbon steel and grey plain cast iron; (**b**) alloy steel and alloy cast iron (based on [17]).

Figure 1 schematically shows two basic variants of SL structure along with their division into individual zones, which are possible to obtain after gas sulfonitriding [16,17].

The complex structure of sulfonitrided layers may have two functions. Firstly, reducing the adhesion affinity of frictional pair elements and diminishing the shear strength of surface irregularities and possible galling. Secondly, it provides increased hardness of the SL, which allows transfer of pressure in the contact zone. Sulfonitrided layers achieved on alloy steels (variant b) are characterized by high hardness in the nitride compounds zone (up to 1300 HV) and in the zone of internal nitriding (from 1200 to 500 HV). Therefore, they can be used in friction nodes loaded with high pressure, sometimes even of linear and point character.

## 2. Materials and Methods

The aim of the experimental program presented in this paper is to assess the impact of commonly used mechanical processes, such as a pre-sulphonitriding treatment, on SL properties of two types of P/M tool steels. Our studies included, in particular:

- Microstructure analysis by scanning and optical microscope,
- X-ray diffraction analysis,
- Micro-hardness measurements of cross-sectional SL,
- 3D surface roughness measurements,
- Evaluation of dynamic friction and wear resistance against Al_2_O_3_ and 19MnB4 steel pins.

The nominal chemical compositions of the two grades are presented in Table 1. The main difference between these materials is the content of C, N, and W. First of all, Vancron 40 contains 1.8% N and 3.7% W, as well as 1.1% C. By contrast, Vanadis 8 is characterized by higher concentrations of C and lack of N and W. The content of other chemical elements is at a similar level.

Samples from both steels were cut and heat treated as indicated in Table 2. The final macrohardness after the heat treatment was 64 ± 1 HRC and 62 ± 1 HRC, respectively, for the Vanadis 8 and Vancron 40 tool steels. Afterwards, two various paths of surface modification were employed:

- Grinding–sulphonitriding (G-SN),
- Hard turning–slide burnishing with 130 N force–sulphonitriding (T-B130-SN).

Front face grinding with cBN (cubic boron nitride) wheels with resinous bond was carried out on a 3E642 type universal tool grinder. The following parameters were used: Peripheral speed of the wheel vs. = 16 m/s; table feed speed vf = 210 mm/min; working engagement ae = 0.02 mm, grit size = B126. Turning process was applied to PCBN (polycrystalline cubic boron nitride) cutting inserts with commercial names NP-DCGW11T302GA2 BC020 by using a constant feed f = 0.07 mm/rev. and the cutting depth ap = 0.2 mm. Cutting speeds were different: vc = 100 m/min for Vanadis 8 and vc = 160 m/min for Vancron 40. Furthermore, slide diamond burnishing of our tool steels in the quenched state was carried out using diamond tools at the following parameters: F = 130 N, f = 0.02 mm/rev., and v = 100 m/min. The applied burnishing force allowed the reduction of surface roughness after turning and the plastic deformation of the surface layer of investigated tool steels in the hardened condition—without abrasive wear of the burnishing ending tip. These tools with tips in the shape of spherical caps were designed, and are currently produced, at our Institute. High pressure–high temperature (HT-HP) Bridgman type apparatus was used to obtain diamond composites with ceramic bonding phase, namely Ti_3_SiC_2_. Compacts were sintered at the pressure of 8.0 ± 0.2 GPa and at 1800 ± 50 °C for 30 s. Subsequently, their spherical shapes were formed by electrical discharge machining (EDM) [18,19].

Finally, after the above-mentioned types of mechanical processes, samples were immediately sulpho-nitrided, under the following conditions: 520 °C/5 h. This technology has been prescribed as one of the operations in the design and manufacturing of tools, dyes, etc.

The X-ray diffraction (XRD) measurements were performed with a PANalytical Empyrean diffractometer (PANalytical B.V., Almelo, Netherlands) using copper radiation (λ_Cu_ = 1.5406 Å). The phase analyses were performed using the ICDD PDF-4 + 2018 files.

Metallographic structures were observed with a scanning electron microscope (JEOL type JSM-6460LV, JEOL Ltd., Akishima, Tokyo, Japan) equipped with an INCA EDS (energy dispersive X-ray spectrometer) and INCA wavelength dispersive spectroscopy (WDS). An optical Carl Zeiss Axiovert 100A microscope (Carl Zeiss AG, Oberkochen, Baden-Württemberg, Deutschland) was also used to observe cross-sections of the SL samples.

Vickers microhardness HV was determined using a FM 7 tester from Future Tech. Corp., Kawasaki, Japan. Microindentations were made using a 100 g load. On each specimen, 3 measurements were performed at various distances below the surface in order to obtain a representative average hardness value.

3D surface roughness parameters were measured using a TOPO 01 laboratory profilometer, developed at our Institute. It was equipped with a 1 mm measuring head, a diamond tip with a radius of 2 μm, and a cone angle of 90°. Data analysis and surface topography were developed in Mountain Map v.7 by Digital Surf.

**Figure 2:**
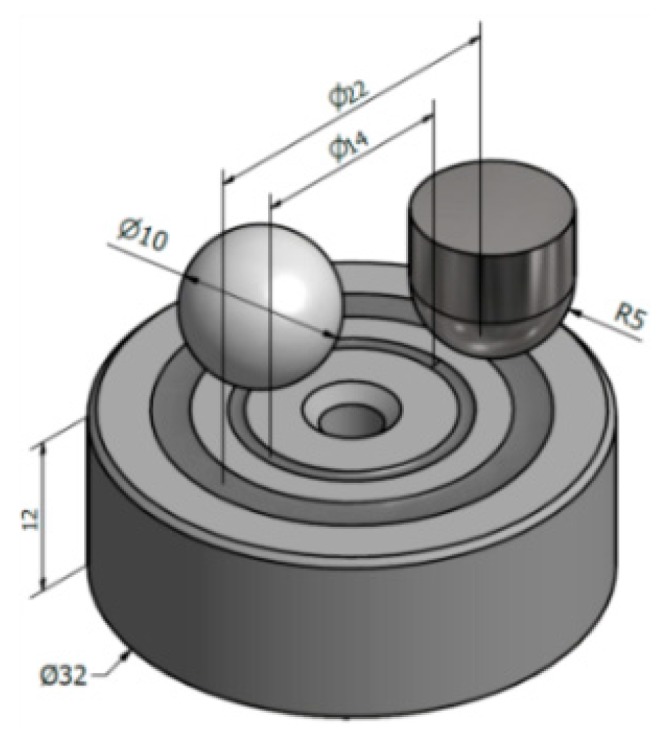
An example of a sample used during the pin-on-disc tests and two types of applied pins as counterbodies; dimensions are given in mm.

The abrasive wear resistance was evaluated by well-known pin-on-disc method [20,21]. More information about the mechanical tester and how the measurements were conducted have been provided in earlier papers [22,23]. The tests were carried out three times without a lubricant at room temperature. Applied test conditions are given in Table 3 and samples are shown in Figure 2. For each sample, two tests using different diameters of the sliding circle 7 mm and 11 mm were made, respectively, for Al_2_O_3_ and 19MnB4 steel pins. It should be noted that for both pins, the same sliding speed was used to avoid a negative impact on the dynamic friction. Moreover, due to the faster wear of steel pins, the shorter sliding distance was used. Dynamic friction µ and wear rate Ws were calculated by well-known standard formulas also reported in [22].

## 3. Results

**Figure 3:**
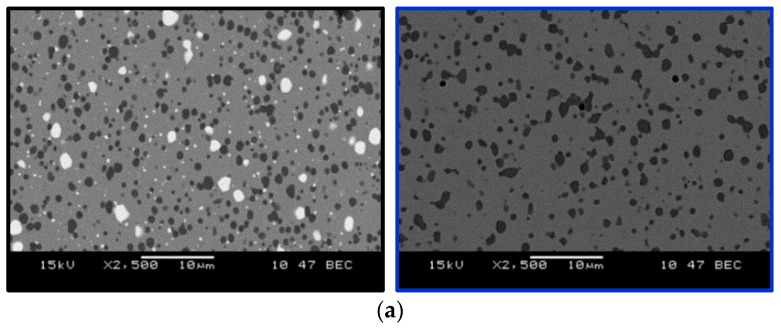

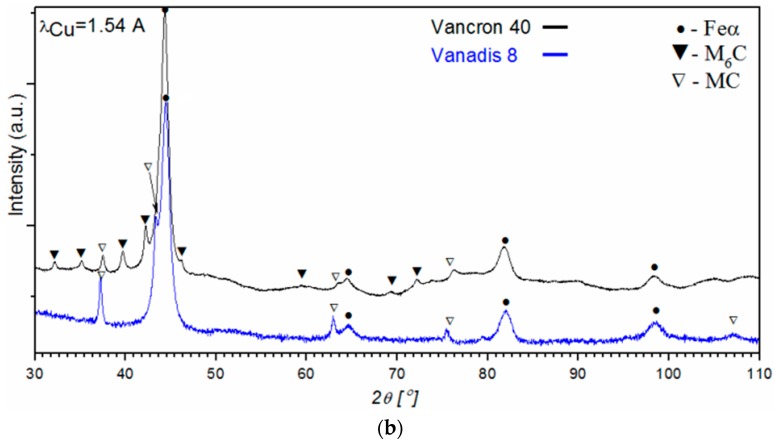
Cross-sectional micrographs (**a**) and results of X-ray diffraction analysis (**b**) for Vancron 40 and Vanadis 8 steels after heat treatment.

In Figure 3a, microstructures of investigated steels after heat treatment are shown: Vancron 40 on the left and Vanadis 8 on the right. In both cases, we see carbide particles within a fine-tempered martensite matrix. Differences are seen in the color of observed carbides. In order to determine their type, X-ray diffraction analysis were performed; see Figure 3b. The black line corresponds to the Vancron 40 steel steel and the microstructure of contains two types of carbides, such as MC and M_6_C, whereas, the blue line corresponds to the Vanadis 8 steel and its microstructure consists only of MC type carbides. According to Jurči [24], excellent wear resistance is ensured by a high amount of carbides, among which the MC particles play a dominant role.

**Figure 4:**
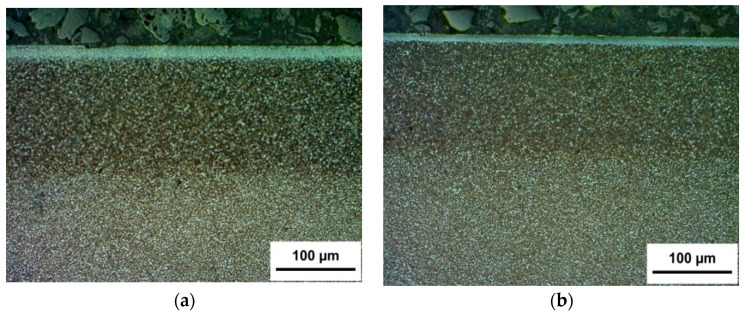
Optical photographs of a cross-section of SL of Vancron 40 steel after: (**a**) Grinding–sulphonitriding (G-SN); (**b**) hard turning–slide burnishing with 130 N force–sulphonitriding (T-B130-SN).

**Figure 5:**
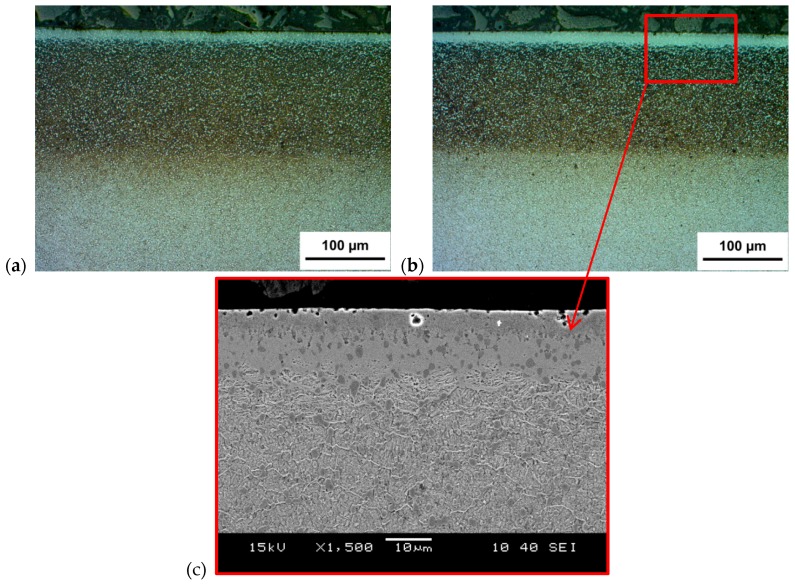
Optical photographs of a cross-section of SL of Vanadis 8 steel after: (**a**) G-SN; (**b**) T-B130-SN; (**c**) image of SL recorded by the scanning electron microscope.

Microstructure changes in the SL of samples after the application of two various paths of surface modification (G-SN and T-B130-SN) were also studied. The optical images of cross-sectional micrographs for both tool steels are presented in Figure 4 and Figure 5. For Vancron 40, the total thickness of the SL comprising the sulfurized zone as well the nitrogen compound zone and a diffusion layer in the G-SN variant reached ≈160 µm (Figure 4a), but for the specimen that had been turned and burnished before sulfonitriding, the thickness of such a layer was slightly smaller (Figure 4b). For Vanadis 8, the corresponding values were very similar; see Figure 5a,b. Meanwhile, the thickness of the sulfurized zone near the surface in case of Vancron 40 steel samples was thicker after G-SN. An opposite effect was found for the Vanadis 8 steel. An example of the etched cross section of the sulfurized and nitrided compound zones recorded on the scanning electron microscope is also provided in Figure 5. Differences in the thickness of sub-surface zones recorded on cross-sections of the surface layer of both steel samples were related to their chemical composition. The difference between C content and elements, such as N and W (see Table 1), could affect the rate of diffusion. Finally, it should be added that after mechanical processes, all samples were subjected to sulphonitriding treatment in one process.

**Figure 6:**
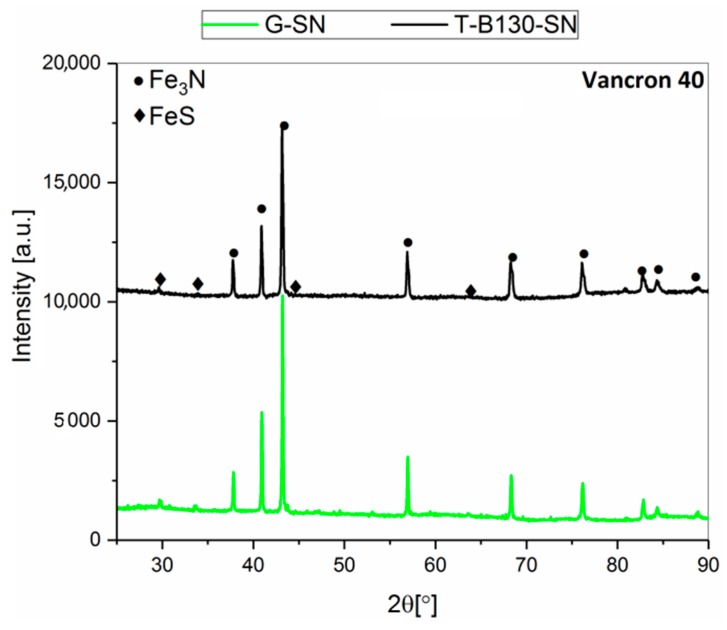
X-ray diffraction spectra recorded in the SL of Vancron 40 tool steel after: G-SN and T-B130-SN.

**Figure 7:**
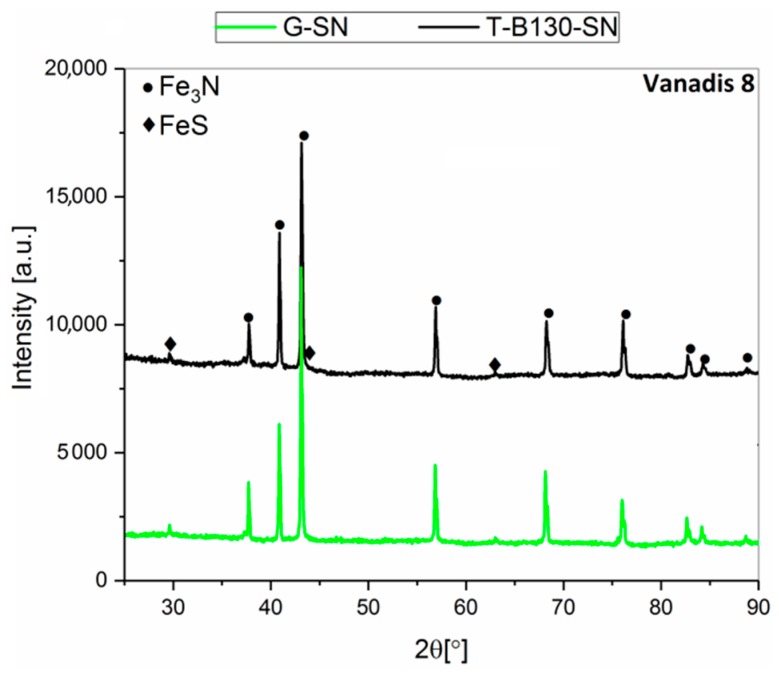
X-ray diffraction spectra recorded in the SL of Vanadis 8 tool steel after: G-SN and T-B130-SN.

Selected results, including X-ray diffraction analysis with phase composition determined in the SL of samples after combined mechanical processes with the chemical heat-treatment operation (sulfonitriding), are presented in Figure 6 and Figure 7 as well as in Table 4 and Table 5. For both steels, the same phases (Fe_3_N, FeS) were found after the two processes applied. No significant differences in their concentrations were found.

**Figure 8:**
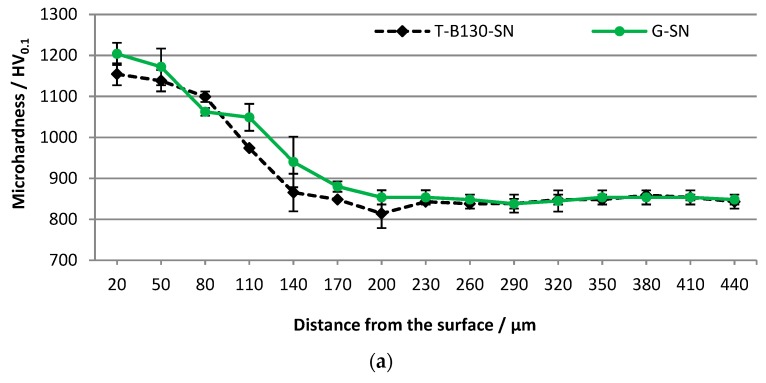

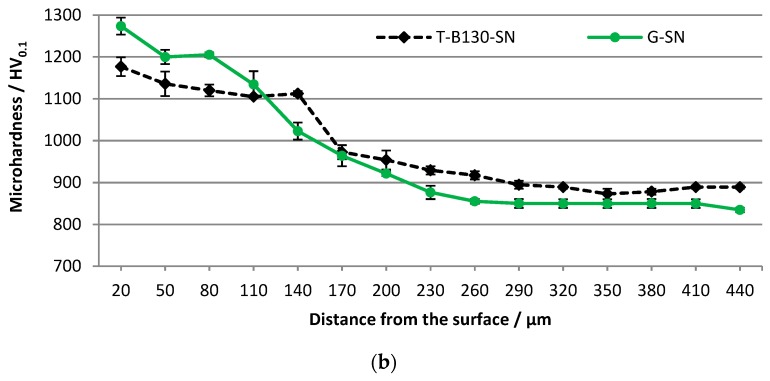
Distribution of microhardness as a function of depth away from the G-SN and T-B130-SN surface for: (**a**) Vancron 40 and (**b**) Vanadis 8 tool steels.

Figure 8 show the microhardness results for the SL of samples previously treated by our two processes. The micro hardness was measured from the surface into the depth of the material on the cross section of the samples. In the case of Vancron 40 steel, it can be seen that slightly higher values (about 5%) were obtained for the SL of samples after the combining process of G-SN. In this variant, the thickness of the upper layer with increased hardness was determined to 230 µm. Only at a distance of 80 μm from the surface, slightly lower values were found. An analogous comparison for Vanadis 8 steel samples indicates that up to a depth of 110 um of SL, a similar trend was found. Meanwhile, deeper towards the core, the opposite effect occurs. Higher values were determined for the samples after the three-stage process.

**Figure 9:**
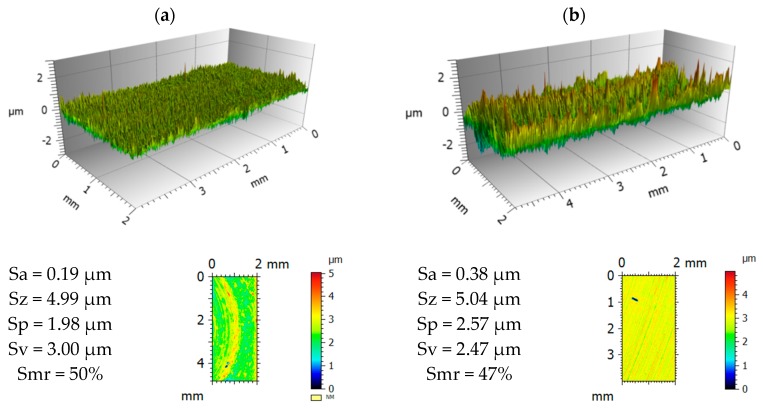
3D view and statistical characteristics of samples surface of Vancron 40 steel after: (**a**) G-SN and (**b**) T-B130-SN.

**Figure 10:**
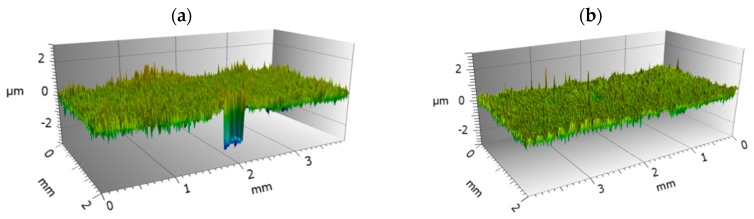

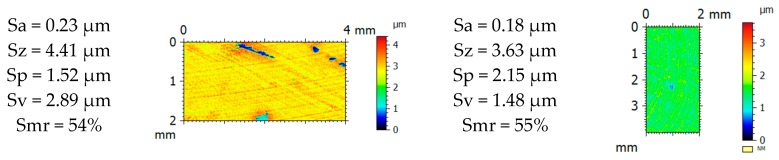
3D view and statistical characteristics of samples surface of Vanadis 8 steel after: (**a**) G-SN and (**b**) T-B130-SN.

3D topographies of sample surfaces for both investigated tool steels are presented in Figure 9 and Figure 10. For Vancron 40 steel samples, lower values of selected parameters were found after the G-SN variant. Comparing the roughness parameter Sa (arithmetical mean height), a two-fold change was found. In connection to the other parameters, the changes are not so significant. In the case of Vanadis 8 steel, slightly lower values were obtained for samples subjected to T-B130-SN treatment.

**Figure 11:**
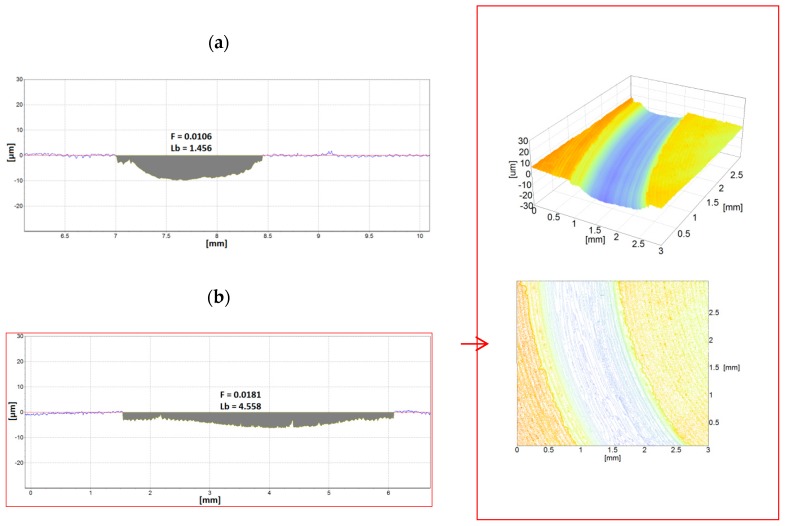
Profiles of wear traces created on Vanadis 8 steel samples (T-B130-SN variant) after tribologicals test using two types of counterbodies: (**a**) Al_2_O_3_; (**b**) 19MnB4; an example of 3D view was added.

Given the nature of the typical wear of tool steels used in cold forming processes, two types of pins as counterbodies were applied for tribological tests. An Al_2_O_3_ ceramic ball was selected to determine abrasion resistance, whereas pins made of low-carbon 19MnB4 steel with the addition of boron were used to determine adhesive resistance. The volume of material removed was significantly different, both for the depth and the width of achieved wear tracks. Example of two profiles of such traces for Vanadis 8 tool steel samples after tests with our types of counterbodies are shown in Figure 11. A 3D view of 3 × 3 mm area is presented for one of them.

**Figure 12:**
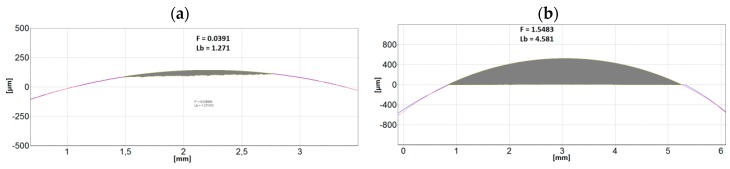
Examples of 2D transverse profiles of pins: (**a**) Al_2_O_3_; (**b**) 19MnB4 steel in the wear trace areas after pin-on-disc tests carried out on Vanadis 40 steel samples (T-B130-SN variant).

Taking into account the differences observed in the wear traces, counterbodies were also evaluated. About 40-fold higher wear of pins made of 19MnB4 steel compared to Al_2_O_3_ ceramic pins was found; examples of such results are shown in Figure 12. It is worth mentioning that the width of wear found on the counterbodies corresponds to the width of wear traces measured on the sample. In the case of ceramic pins, the width is ≈1.3 mm, whereas for steel pins, it amounts to more than 4 mm.

**Figure 13:**
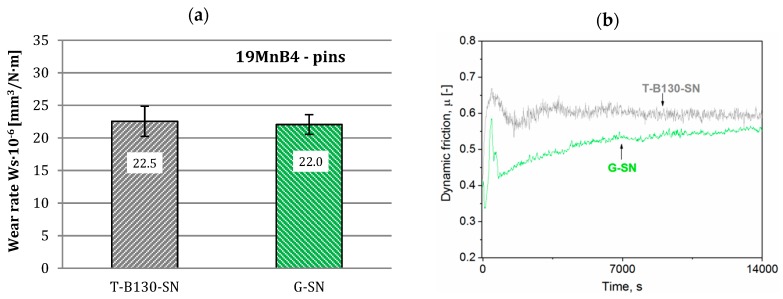
Wear rates (**a**) and dynamic friction (**b**) values for Vancron 40 steel after G-SN and T-B130-SN; 19MnB4 steel pins as counterbodies were used.

**Figure 14:**
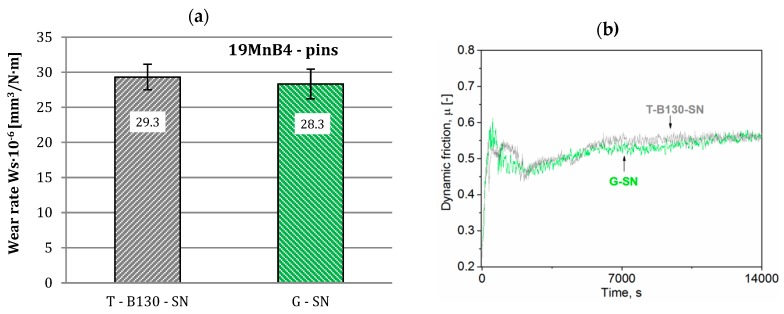
Wear rates (**a**) and dynamic friction (**b**) values for Vanadis 8 steel after G-SN and T-B130-SN; 19MnB4 steel pins as counterbodies were used.

Wear rates and dynamic friction values determined for our steels after pin-on-disc tests against 19MnB4 pins are shown in Figure 13 and Figure 14. It should be noted that minor changes in wear rates for samples subjected to different processes of SL modification are found; this applies to both steels. Differences between T-B130-SN and G-SN variants amount to 2% and 3%, respectively, for Vancron 40 and Vanadis 8 steels. This is related to the much lower hardness of the 19MnB4 steel pin relative to the sample. Taking into account results of dynamic friction for samples of Vancron 40 steel, lower values were observed after G-SN variant; see Figure 13b. The 2-fold higher surface roughness of samples (Figure 9b) may explain such a result. For Vanadis 8 steel samples, a very similar trend and values of dynamic friction are observed. In case of this steel, similar surface topography was obtained after both variants of surface modification were applied.

**Figure 15:**
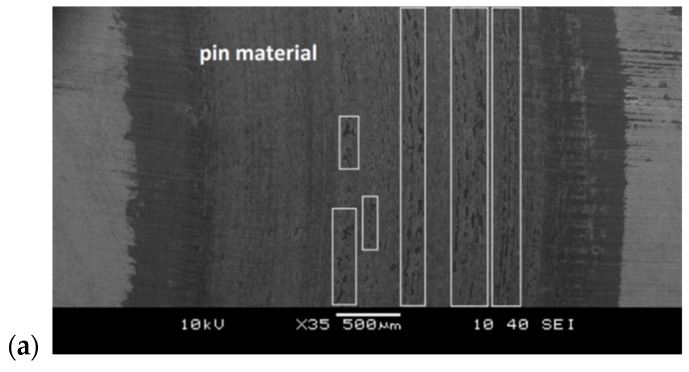

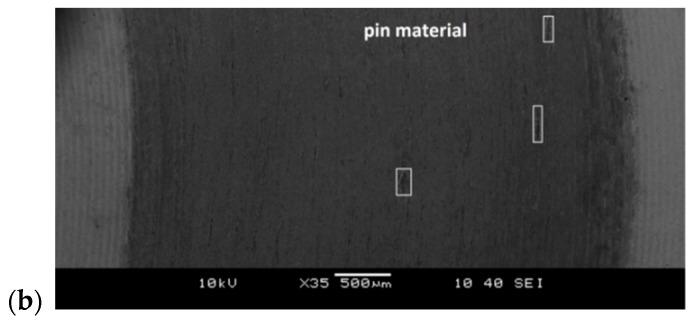
SEM images of wear traces created after pin-on-disc tests using 19MnB4 steel pins on Vanadis 8 steel samples, subjected to selected variants of surface treatment: (**a**) G-SN and (**b**) T-B130-SN.

Deposits of pin material on the sample’s surface were the dominating features on the wear tracks created after pin-on-disc tests using 19MnB4 steel pins. Examples of SEM images of wear traces on Vanadis 8 steel samples are shown in Figure 15. Large deposits of material built up on the disc surface in the G-SN variant appeared. Only a little transfer of pin material on the sample surface was observed after three stage process. Unlubricated sliding wear tests of D2 steel (sample) and AISI 1018 steel (pin) pairs with more than a twice higher hardness of samples, carried out by Okonkwo et al. [25], indicate that the material adhesion was also the dominant mechanism for the ambient temperature wear tests.

**Figure 16:**
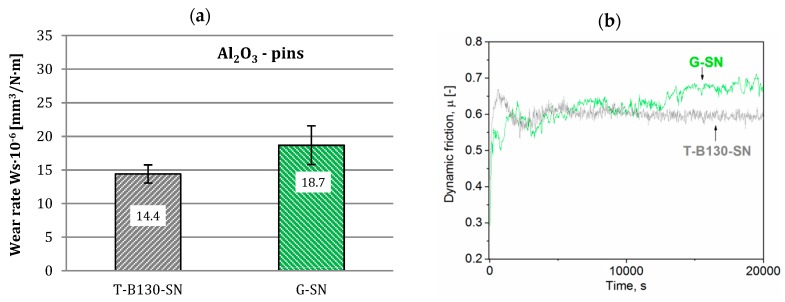
Wear rates (**a**) and dynamic friction (**b**) values for Vancron 40 steel after G-SN and T-B130-SN; Al_2_O_3_ pins as counterbodies were used.

**Figure 17:**
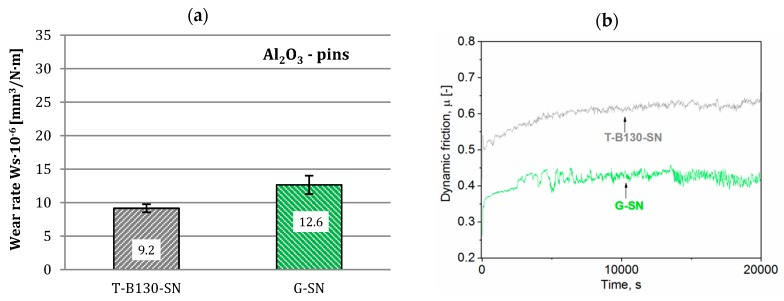
Wear rates (**a**) and dynamic friction (**b**) values for Vanadis 8 steel after G-SN and T-B130-SN; Al_2_O_3_ pins as counterbodies were used.

On the other hand, wear rates and dynamic friction values determined for Al_2_O_3_ pins are shown in Figure 16 and Figure 17. Application of the T-B130-SN variant was beneficial for abrasion resistance of both steels. A significant increase in wear resistance has been achieved compared to the G-SN variant. It reached 30% and 37%, respectively, for Vancron 40 and Vanadis 8 tool steels. Moreover, a simple comparison between examined steels subjected to three-stage process points to more than 50% increase of abrasion wear resistance of Vanadis 8 steel. The type of carbides in the microstructure is undoubtedly one of the explanations for these results. Compressive residual stress is another factor that could affect such a result. According to previous studies, a homogenization of stress state to the isotropic (σ_1_ = σ_2_) compression after application of the sequential technology (e.g., turning–burnishing–nitriding) was observed, contributing to increased wear resistance. More information on this subject can be found in an earlier paper [26].

**Figure 18:**
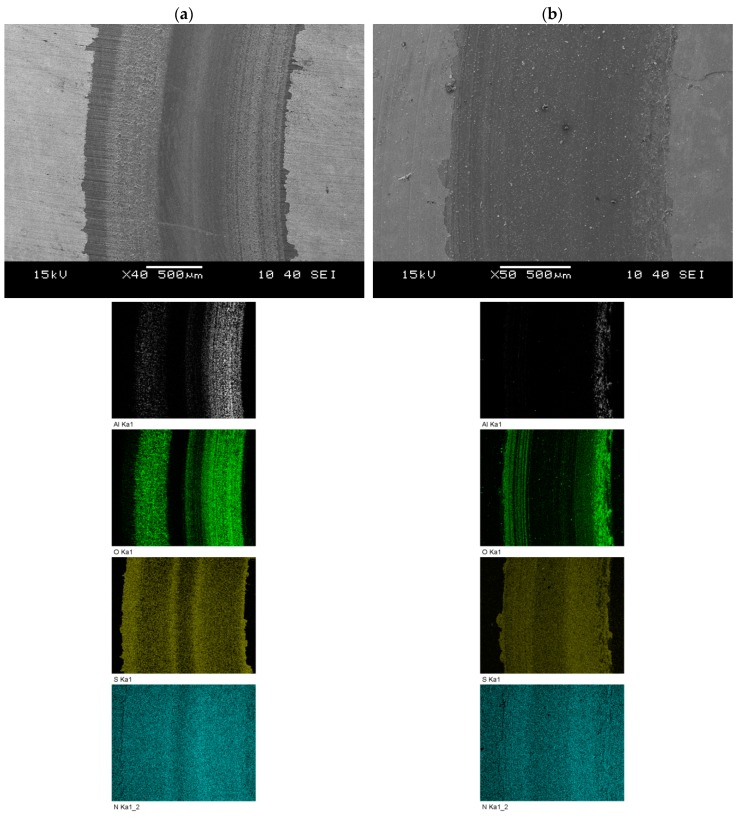
SEM images of wear traces (with energy dispersive X-ray spectrometer (EDS) mapping analysis) created after the tribological test using the pin-on-disc method on Vanadis 8 steel samples, subjected to selected variants of surface treatment: (**a**) G-SN and (**b**) T-B130-SN.

Finally, SEM micrographs of wear tracks created in pin-on-disc tests against Al_2_O_3_ pins for samples of Vanadis 8 steel are shown in Figure 18. Images and chemical analysis reveal a much smaller deposition of pin material on the sample surface in the case of T-B130-SN variant. Taking into account the main wear mechanisms, the abrasive wear dominates in this case. Furthermore, some differences of S and N content in the wear tracks were observed—it is related to their depth created after tests.

## 4. Conclusions

The following conclusions can be formulated:

- Type of machining as a pre-sulphonitriding treatment is very important for SL properties of hardened P/M tool steels, such as microhardness or wear resistance.
- There were no significant differences in wear rates between investigated processes after pin-on-disc tests with 19MnB4 steel pins, whereas several times lower transfer of pin material on the samples surface subjected to T-B-SN variant compared to G-SN was observed. Micro adhesive tacks welding are visible as matte areas on wear tracks along the sample surfaces, in the G-SN variant.
- Application of the three-stage process was also beneficial for abrasion resistance after tests with Al_2_O_3_ pins. At least a 30% increase in wear resistance has been achieved for both investigated steels.
- Furthermore, for samples subjected to the three-stage process, more than a 50% increase of wear resistance (against Al_2_O_3_ pins) was obtained for Vanadis 8 steel compared to Vancron 40. Different microhardness of carbides occurring in the microstructure (from, in HV 0.02, ≈3000 for MC type to ≈1500 for M_6_C) after heat treatment was also a large influence on the wear resistance of both tool steels.
- Finally, the three-stage process can be an alternative to G-SN considering two types of wear (adhesion or abrasion).

## Figures and Tables

**Table 1:** Chemical compositions of tool steels (wt. %).

Steel	C	N	Si	Mn	Cr	Mo	W	V
Vanadis 8	2.3	-	0.4	0.4	4.8	3.6	-	8.0
Vancron 40	1.1	1.8	0.5	0.4	4.5	3.2	3.7	8.5

**Table 2:** Heat treatment parameters for Vanadis 8 and Vancron 40 tool steels.

Stages of Heat Treatment	Vanadis 8	Vancron 40
Austenitizing	1090 °C	1030 °C
First tempering	560 °C, 2 h	550 °C, 2 h
Second tempering	560 °C, 2 h	550 °C, 2 h
Third tempering	560 °C, 2 h	550 °C, 2 h

**Table 3:** Conditions for tribological tests carried out on the Vanadis 8 and Vancron 40 steel samples.

Pin Material	Hardness, HV	Diameter of the Sliding Circle r/mm	Sliding Distance L, m	Applied Load F, N	Sliding Speed v, m/s
Al_2_O_3_	1800	7.0	2000	25	0.1
19MnB4 Steel	385	11.0	1400		

**Table 4:** Phase composition in the SL of Vancron 40 tool steel after: G-SN and T-B130-SN.

Phase Composition	% mas.	
	G-SN	T-B130-SN
Fe_3_S	99	≈100
FeS	1	<1

**Table 5:** Phase composition in the SL of Vanadis 8 tool steel after: G-SN and T-B130-SN.

Phase Composition	% mas.	
	G-SN	T-B130-SN
Fe_3_S	99	99
FeS	1	1
